# Assessment of Sustainable Elimination Criteria for Iodine Deficiency Disorders Recommended by International Organizations

**DOI:** 10.3389/fnut.2022.852398

**Published:** 2022-04-13

**Authors:** Lijun Fan, Fangang Meng, Qihao Sun, Yuqian Zhai, Peng Liu

**Affiliations:** ^1^National Health Commission and Education Bureau of Heilongjiang Province, Key Laboratory of Etiology and Epidemiology, Harbin Medical University, Harbin, China; ^2^Heilongjiang Provincial Key Laboratory of Trace Elements and Human Health, Harbin Medical University, Harbin, China; ^3^Endemic Disease Control Center, Chinese Center for Disease Control and Prevention, Harbin Medical University, Harbin, China

**Keywords:** iodine deficiency disorders, urine iodine concentration, goiter prevalence, iodized salt, sustainable elimination

## Abstract

Enormous efforts have been made to evaluate the worldwide prevention and control of iodine deficiency disorders (IDDs). This study evaluated China's achievements in IDD prevention and control against WHO criteria for sustainable elimination of IDD. The study sample consisted of 556,390 school-aged children and 271,935 pregnant women enrolled in the 2018 China National IDD Surveillance. As a result, at the national level, median urine iodine concentration (MUIC) was 206.1 and 163.5 μg/l in children and in pregnant women, respectively. The proportion of households consuming adequate iodized salt (PHCAIS) was 90.2%. The prevalence rates of goiter in children and thyroid disease in pregnant women were 2.0 and 0.8%, respectively. MUIC showed significant non-linear increasing trends with increasing PHCAIS in both children and pregnant women. The prevalence of thyroid disease in pregnant women had a sharp decreasing trend with increasing PHCAIS. Of note, the prevalence of goiter in children and thyroid disease in pregnant women against MUIC both presented as significant U-shaped curves, with the lowest prevalence at 100–300 μg/l of MUIC in children and 150–250 μg/l in pregnant women. PHCAIS, MUIC, and the programmatic indicators at the national level were all above their cut-offs proposed in the 2007 Criteria. Evaluation by adding the prevalence of goiter (<5%) yielded the different results at the county level. Sustainable elimination of IDD has been achieved nationally. 2018 Chinese surveillance data support the expansion of global cut-offs for optimal iodine status in school-age children from 100–199 to 100–299 μg/l as recommended by others and the lower limit of MUIC (150 μg/l) in pregnant women also seems justified. Inclusion of goiter prevalence <5% in our analysis reduced the number of municipalities and counties which had achieved sustainable elimination of IDD.

## Introduction

Iodine is an essential element required to produce thyroid hormones and plays a major role in human growth and tissue development (1). It is well-known that reduced iodine intake is associated with iodine deficiency disorders (IDDs), namely, endemic goiter, hypothyroidism, and cretinism (2), a decreased fertility rate, an increased incidence of perinatal death, and infant mortality (3).

In the 1990s, the WHO recommended universal salt iodization (USI) as the first-line strategy for IDD prevention and control (4, 5). The WHO, the United Nations International Children's Emergency Fund, and the International Council for Control of Iodine Deficiency Disorders (currently the Iodine Global Network) developed the guideline “Assessment of iodine deficiency disorders and monitoring their elimination: A guide for program managers, 3rd edition.” Chapter 6 of this guide on “Indicators of the sustainable elimination of IDD” includes “technical criteria” for the sustainable elimination of IDD with regards to salt iodization and population iodine status (Table 10) and 10 programmatic indicators, hereafter referred to as the “2007 criteria” (2).

The USI has been implemented in China for over two decades. The latest Chinese surveillance data showed that iodine nutritional status of the general population and pregnant women has been greatly improved (6). A series of programmatic indicators which are similar to those in the 2007 criteria were established in China. By 2000, IDD had been essentially eliminated in China (7).

Since the publication of the 2007 guideline “Assessment of iodine deficiency disorders and monitoring their elimination: A guide for program managers, 3rd edition,” expansion of the range of median of urinary iodine concentration (MUIC) considered optimal in school-age children has been suggested (8, 9) and the necessity to achieve >90% proportion of households consuming adequate iodized salt (PHCAIS) has been questioned (10). This article also suggests the inclusion of the prevalence of goiter >5% as criteria against which to assess the sustainable elimination of IDD. At present, considerable efforts have been made worldwide to evaluate the effect of USI on the sustainable elimination of IDD. Therefore, the revision and application of the 2007 criteria are more important than ever.

This study aims to assess the achievement of the sustainable elimination of IDD in China based on the technical and programmatic indicators in the 2007 criteria and the Chinese criteria and to provide evidence for revision of the guidelines. This aim was achieved by analyzing the 2018 China National IDD Surveillance data (6).

## Methods

### Data Source and Sampling Method

In the 2018 China National IDD Surveillance, a multistage sampling procedure was used with the county being the sampling unit. Each county was divided into five sampling areas: east, west, south, north, and middle, and one town or district was randomly sampled from each area. Out of 2,879 counties, we analyzed data from 2,827 counties having water iodine concentration (WIC) <100 μg/l, with 52 counties having WIC ≥ 100 μg/l excluded.

Children of ages 8–10 years (*n* = 40) from local primary schools and pregnant women (*n* = 20) from communities around the schools were randomly selected from each of the five sampling areas in each county. A total of 200 children and 100 pregnant women were recruited from each county. The inclusion criteria were as follows: residents living in the sampling site for at least half a year; an equal number of girls and boys; pregnant women who were diagnosed as being pregnant in a medical institution.

Information on geographic regions, coastal living, and capita income was collected from the State Statistical Bureau. Information on date of birth, sex, ethnicity, gestational weeks, and history of thyroid disease (including thyroid nodules, clinical and subclinical hyperthyroidism, clinical and subclinical hypothyroidism, thyroiditis, and thyroid cancer) was obtained using a questionnaire. Spot urine specimens were collected from all participants, and salt samples were collected from their households. We also analyzed the data from the National Survey on Iodine in Drinking Water (11). WIC at the county level was used to establish the inclusion criteria for the current study.

The study protocol was approved by the ethics committee of the Harbin Medical University. Written informed consent was obtained from all participants or their guardians.

### Laboratory Analysis

The direct iodine titration method was used to assay the iodine content of salt samples (12). Salt consumed was classified as non-, low-, qualified, and high-iodized salt according to the iodine contents as shown in Supplementary Table 1. In China, provinces are allowed to select one/two of three options for salt iodine content—20, 25, or 30 mg/kg with a variation of ±30%, according to the Chinese standard (GB 26878) (13). At present, 14 provinces selected 25 (14–29) mg/kg, 12 provinces selected 30 (17–35) mg/kg, and 5 provinces selected 25 mg/kg for the general population and 30 mg/kg for pregnant women (13). As^3−^-Ce^4+^ catalytic spectrophotometry was used to measure urinary iodine concentration (36), and the iodine nutritional status at different levels was evaluated according to the international standard (2). The thyroid volume of children was measured using B-ultrasonography. Goiter was diagnosed as thyroid volume being above the Chinese standard (37).

### Assessment of Elimination of IDD

The 2018 China National IDD Surveillance data have four levels, i.e., national, provincial, municipal, and county. Then for each level, elimination of IDD was assessed based on individual technical indicators and multiple technical indicators in 2007 criteria plus programmatic indicators in both the 2007 criteria and the Chinese criteria. Chinese criteria for evaluating the sustainable elimination of IDD (a scoring table) are shown in Supplementary Table 2. Individual items were scored based on IDD elimination achievements in the past 3 years, and then individual scores were added up as a total score. Areas with a total score ≥ 85 along with relevant technical indicators were classified as sustainable elimination of IDD.

### Statistical Analysis

All the survey data were entered into the IDD Information Management System (a standard data management system). Statistical analyses were performed using SAS version 9.1 (SAS Institute, Incorporation, Cary, North Carolina, USA). The differences in continuous and categorical variables between groups were tested using the ANOVA/Wilcoxon's test and the chi-squared test, respectively. The curve between MUIC and PHCAIS in children and pregnant women and the curve of MUIC with the prevalence of goiter in children and prevalence of thyroid disease in pregnant women were fitted by polynomial regression analysis models.

## Results

### Descriptive Data

Descriptive data of the study sample are given in Table 1. The variables showed significant increasing or decreasing trends across most subgroups. In the total sample of the 2018 China National IDD Surveillance, MUIC was 206.1 and 163.5 μg/l in children and in pregnant women, respectively; PHCAIS was 90.2%; the prevalence rates of goiter in children and thyroid disease in pregnant women were 2.0 and 0.8%, respectively.

**Table 1:** Descriptive data of the study sample.

**Variable**	**Children**						**Pregnant women**			
	**TV (mL)**		**Goiter prevalence**		**MUIC (μg/L)**		**TD prevalence**		**MUIC (μg/L)**	
	***N***	**Median**	***N***	**(%)**	***N***	**Median**	***N***	**(%)**	***N***	**Median**
**Children age (year)**										
8	70,110	2.41^*^	70,110	2.15^*^	150,914	203.10^*^	—	—	—	—
9	100,793	2.60	100,793	1.96	217,410	205.20	—	—	—	—
10	87,701	2.84	87,701	1.27	188,006	209.70	—	—	—	—
**Pregnant stage**
1st-	—	—	—	—	—	—	537	0.94^*^	56,306	166.30^*^
2nd-	—	—	—	—	—	—	926	0.79	115,577	165.20
3rd-	—	—	—	—	—	—	772	0.77	100,052	159.20
**Type of iodized salt**
Non-iodized	8,678	2.91^*^	8,678	3.09^*^	18,895	163.10^*^	238	2.98^*^	7,972	125.59^*^
Low-iodized	10,772	2.68	10,772	2.40	24,239	195.30	79	0.85	9,270	158.00
Qualified iodized	240,571	2.61	240,571	1.70	510,150	198.40	1,566	0.76	206,245	166.50
High-iodized	2,613	2.65	2,613	1.95	5,187	214.70	—	—	2,274	164.34
**Water iodine (μg/L)**
<10	232,348	2.60^*^	232,348	1.72	482,599	194.50^*^	1,968	0.83	237,588	161.60^*^
10~	26,841	2.71	26,841	2.16	57,010	205.90	248	0.83	29,619	168.00
40~	2,210	2.86	2,210	3.53	9,998	251.25	—	—	3,489	206.00
100~	3,248	2.90	3,248	2.00	6,723	281.60	—	—	3,291	223.53
**Coastal region**
Yes	20,546	2.63^*^	20,546	1.81^*^	36,774	174.50^*^	453	2.47^*^	18,116	131.19^*^
No	160,519	2.61	160,519	1.72	349,317	199.03	1,278	0.74	172,804	165.60
**Landform**
Plain	62,523	2.69^*^	62,523	2.11^*^	135,671	197.80^*^	753	1.11^*^	67,640	162.50
Hilly	48,006	2.58	48,006	1.61	113,612	196.10	556	0.99	55,920	159.94
Mountain	66,293	2.57	66,293	1.51	126,975	196.10	382	0.61	62,390	165.40
**Capita income**
< ¥20,000	170,756	2.63	170,756	1.70^*^	260,690	197.10	1,009	0.56^*^	178,258	165.50^*^
¥20,000–¥40,000	73,755	2.66	73,755	1.91	153,655	197.91	725	0.80	75,696	160.30
>¥40,000	20,136	2.64	20,136	2.13	41,232	197.41	501	2.49	20,033	152.40
**Nationwide**	264,740	2.61	264,740	2.00	570,271	206.10	274,929	0.80	274,578	163.50

*TV, thyroid volume; MUIC, median urine iodine concentration; TD, thyroid disease*;

* *p < 0.05 for trend*.

### Associations Between PHCAIS, MUIC, and Thyroid Disease

**Figure 1:**
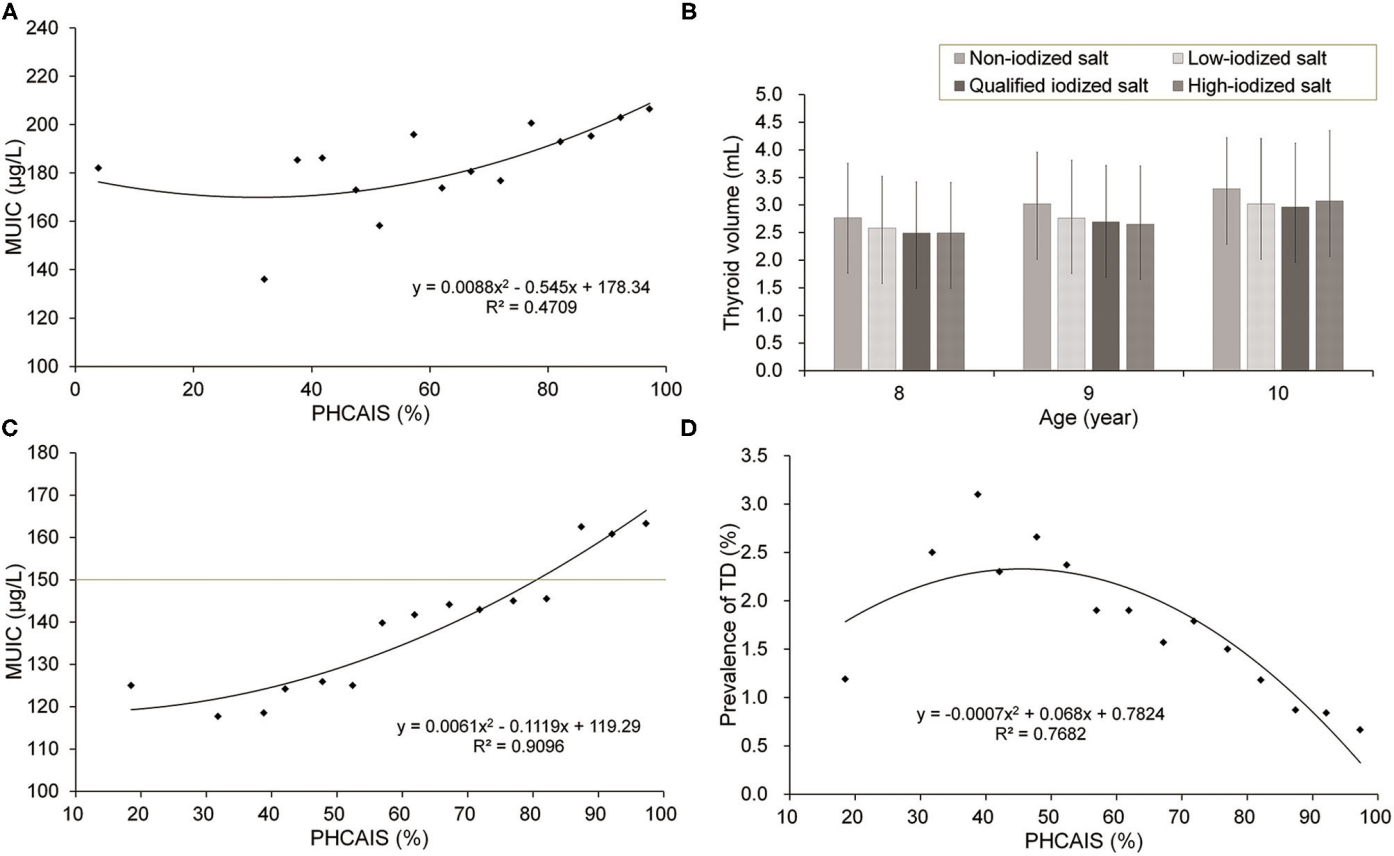
The influence of iodized salt on thyroid disease and iodine nutrition. MUIC, median urine iodine concentration; PHCAIS, proportion of households consuming adequately iodized salt; TD, thyroid disease. **(A,B)** children and **(C,D)** pregnant women. *p* <0.0001 for trend in all 3 age groups in **(B)**.

In Figure 1, PHCAIS was divided into the 15 groups (<30% and groups with an interval of 5% from 30% onward) for Figures 1A,C,D. There were significant non-linear increasing trends of MUIC with increasing PHCAIS in children (Figure 1A) and pregnant women (Figure 1C). It is notable that MUIC of school-age children was > 130 μg/l in all the PHCAIS groups, including those <90% and even those groups <40%. This implies sources of iodine other than household salt. The prevalence of thyroid disease in pregnant women slightly increased and then sharply decreased with increasing PHCAIS (Figure 1D). In Figure 1B, thyroid volume showed significant and consistent decreasing trends across the four subgroups consuming different types of iodized salt within age groups (*p* <0.0001 for the trend in all three age groups). The thyroid volume was the smallest in the qualified iodized salt group in age groups of 8 and 10 years and the largest in the non-iodized salt group for all the age groups.

**Figure 2:**
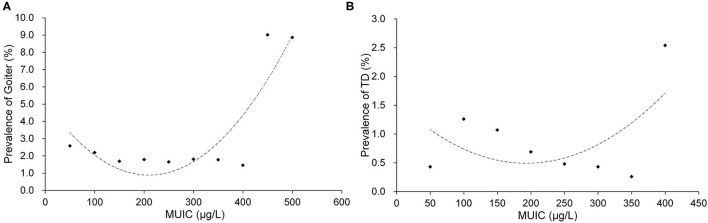
The relationships of MUIC with goiter in children **(A)** and TD in pregnant women **(B)**. MUIC, median urine iodine concentration; TD, thyroid disease. **(A)** *R*^2^ = 0.7763 and **(B)** *R*^2^ = 0.3059.

Figure 2 presents the relationships of MUIC with goiter in children (Figure 2A) and thyroid disease in pregnant women (Figure 2B). MUIC was divided into 10 groups (<100 μg/l, groups with an interval of 50 and ≥500 μg/l) in Figure 2A and 8 groups (<100 μg/l, groups with an interval of 50 and ≥ 400 μg/l) in Figure 2B. The prevalence rates of goiter in children and thyroid disease in pregnant women both showed a significant U-shaped tendency with increasing MUIC. The prevalence of goiter in children (Figure 2A) and the prevalence of thyroid disease in pregnant women (Figure 2B) were the lowest around 200 μg/l of MUIC.

### Evaluation of Sustainable Elimination of IDD in China

Achievement of the sustainable elimination of IDD in China was assessed by four different methods using the 2018 China National IDD Surveillance data, as shown in Table 2. The rates at the national level were 100%. The rates by methods 1 and 2 were higher than those by methods 3 and 4 (53.0 vs. 51.4% at the municipal level and 44.5 vs. 41.9% at the county level). When more indicators were used for evaluation (method 3, 4), the rates of elimination of IDD became lower at the municipal and county levels.

**Table 2:** Evaluation results of IDD in China.

**Unit**	**Total**	**Number of units assessed by individual indicators**				**Rates**, ***n*** **(%)**			
		**PHCAIS** **(>90%)**	**MUIC^**a**^** **(100–299 μg/L)**	**MUIC^**b**^** **(150–249 μg/L)**	**GP** **(<5%)**	**Method 1**	**Method 2**	**Method 3**	**Method 4**
Nation	1	1	1	1	1	1 (100.0)	1 (100.0)	1 (100.0)	1 (100.0)
Province	31	22	31	22	31	17 (54.8)	17 (54.8)	17 (54.8)	17 (54.8)
Municipality	366	280	359	245	350	194 (53.0)	194 (53.0)	188 (51.4)	188 (51.4)
County	2,827	2,125	2,700	1,717	2,639	1,257 (44.5)	1,257 (44.5)	1,185 (41.9)	1,184 (41.9)

*PHCAIS, proportion of households consuming adequately iodized salt; MUIC, median urine iodine concentration; GP, goiter prevalence*.

a *Children and*

b *pregnant women*.

*Method 1 represents the results evaluated by technical indicators of PHCAIS (>90%), MUIC (100–299 μg/l) in children and MUIC (150–249 μg/l) in pregnant women in the 2007 criteria*.

*Method 2 represents the results evaluated by the indicators in Method 1 plus the programmatic indicators in the 2007 criteria*.

*Method 3 represents the results evaluated by the indicators in Method 1 plus GP < 5%*.

*Method 4 represents the results evaluated by the indicators in Method 3 plus a score >85 of programmatic indicators in the Chinese criteria*.

*Rate (%) = the number of areas meeting the criteria/total number of areas × 100*.

## Discussion

In this study, we assessed the IDD elimination status in China of 2018 based on the 2007 criteria and the Chinese criteria using the 2018 China National IDD Surveillance data. Since China has a vast territory with a huge population, we evaluated the rates of IDD elimination at various levels from nation to the county to examine variation in the attainment of IDD elimination among areas and regions. The 2018 China National IDD Surveillance provided an excellent opportunity to evaluate the IDD elimination status in China. The 2018 surveillance system covers all counties in China and has a huge sample size with data available on thyroid volume in school-age children and a history of thyroid disease in pregnant women. Therefore, we took these advantages to conduct comprehensive analyses to assess the IDD elimination status in China for the purpose of providing convincing evidence for the revision of current international and domestic guidelines.

Goiter has been recognized as a historical indicator for assessing the severity of IDD (38). A prevalence of goiter ≥5% in children is a signal of a public health problem (2). Instead of adults, children have a very low prevalence of autoimmune thyroiditis and other thyroid disease (14, 39). Besides, the cutoff point of 5% allows both for some margin of error of goiter assessment, and for goiter that may occur in iodine-replete populations due to other causes such as goitrogens and autoimmune thyroid diseases based on the WHO's guideline (2). However, the prevalence of goiter was not included as an indicator in the 2007 criteria (2). The major reason might be concerns regarding limitations of B-ultrasound (high cost, electricity usage, and requirement for special training) 14 years ago, especially in some developing countries and regions (15, 16). The results from this study showed that method 3 (the 2007 criteria + prevalence of goiter <5%) identified 72 counties less than method 1 (the 2007 criteria alone) in evaluating the elimination (Table 2). The difference in the number of counties meant that these 72 counties might be at high risk for IDD due to insufficient iodine intake that resulted in a higher prevalence of goiter. Based on the observation of this study, the inclusion of the prevalence of goiter <5% as a technical indicator is recommended for the revision of the 2007 criteria. Furthermore, a goiter prevalence ≥5% in school-aged children showed as a signal of a public health problem (2), while a goiter prevalence <5% reflects a long-term endeavor to reduce IDD prevalence in a country. Under a long-term intervention of USI, goiter prevalence should be <5% in all areas. Once goiter prevalence rises to more than 5% in an area, this area might be a high-risk area that may suffer IDD again. Therefore, although goiter prevalence might be not a timely indicator for “iodine nutrition status,” it should still be an indicator for the inadequacy of “IDD prevention and control measures” for a period past, which identified as to whether the prevention measures have been “sustained” in place, correspondence to “sustained” elimination. Furthermore, at the individual level, thyroid volume changes inversely in response to alterations in iodine intake, with a lag interval that varies between a few months and several years. However, goiter prevalence is a population indicator, and because of the sampling method, participants for each year's surveillance are different. It means if the persons in one group consume iodized salt more than 50%, MUIC will be appropriate. However, even 5% of people consume non-iodized salt for more than 6 months, goiter prevalence can be more than 5%. Therefore, goiter prevalence is even more sensitive than MUIC when iodine deficiency, as only a few individuals present goiter, it could be caught by the surveillance under the long-term USI surroundings. Besides, although ultrasound scan is a precise, safe, and non-invasive measurement of thyroid volume and the limitations of B-ultrasound have been substantially reduced, along with the improvement in the economy worldwide, it may still not be feasible in some countries and regions due to the poverty, and in these areas, it can be replaced by palpation. Goiter examination by palpation is not as accurate as by B-ultrasound, however, the error could be lessened by training. Chinese IDD surveillance data (17–19) also showed the results by palpation were acceptable (Chinese IDD surveillance data from 1997 to 2002, *r* = 0.88, *p* <0.0001; shown as Supplementary Figure 1). Therefore, we suggested that under extreme conditions, when thyroid volume assessment using B-ultrasound is not feasible, it might be replaced by palpation.

The proportion of households consuming adequate iodized salt (PHCAIS) (>90%) is one of the technical indicators proposed in the 2007 criteria (2). However, a previous study reported that MUIC in children tended to be adequate, and prevalence of IDD was low in some areas with PHCAIS <90% in China, and then a question was raised, i.e., whether it is necessary to require PHCAIS > 90% in these areas (10). We found in this study that with the increase of PHCAIS, MUIC in both children and pregnant women showed an upward trend (Figures 1A,C). And with increasing PHCAIS, the prevalence of thyroid disease in pregnant women showed a sharp decrease (Figure 1D). The association between PHCAIS and MUIC indicates that PHCAIS is a key factor to maintain iodine nutritional health and a crucial indicator for the evaluation of IDD elimination. Although MUIC in children was adequate in subgroups with PHCAIS <90%, MUIC in pregnant women was below 150 μg/l in almost all these subgroups, suggesting that PHCAIS > 90% is necessary to ensure adequate iodine nutrition levels, especially for pregnant women.

It showed that as well as the lowest thyroid volume, school-age children who consumed qualified and higher iodized salt had the highest MUIC values (Table 1 and Figure 1B), which proved that intake of qualified iodized salt is very important. However, it should also be noted that MUIC in children was higher than 130 μg/l in all subgroups including those with PHCAIS <90% in this study (Figure 1A) and children who consumed non-iodized salt still had adequate iodine status (Table 1). This phenomenon was also seen in the Chinese population in samples/cohorts research that was different from ours (10, 20, 21). Multiple possible reasons may be responsible for these observations. First, part of school-aged children has lunch in school dining rooms where iodized salt is strictly required for cooking. However, the salt in this survey only came from children's households. This might contribute in part to the high MUIC in children in the counties with low PHCAIS in China. Second, other dietary sources of iodine such as milk, eggs, and processed foods containing added iodized salt may account for a proportion of total iodine intake in children. A study on processed food (including instant noodles, biscuits, smoked meat, puffed food, canned meat, milk products, etc.) was performed in six provinces in China, and the results showed that processed foods accounted for 4.0% (10.9 μg/d) of the total iodine intake (22). Third, PHCAIS is defined as the proportion of households consuming “qualified” salt, not including high-iodized salt. Children who consumed high-iodized salt were also classified in the subgroups with low PHCAIS (<90%). Although the situation of iodine intake from school dining room and processed food might be special in China, concerning global status, in this study, it was suggested that proportion of food cooked in school dining rooms and processed food made with iodized salt should be considered as supplementary items in the surveillance to define PHCAIS, especially in urban areas where people more often dine out and have more intake of iodine from the diet such as processed food (23, 24).

We found in this study that the prevalence of goiter in children presented a marked U-shaped relationship with MUIC, with the prevalence being the lowest around 200 μg/l of MUIC (Figure 2A). This observation is consistent with the findings in children from previous studies (25–27). Furthermore, the prevalence of thyroid disease in pregnant women also showed a substantially similar U-shaped relationship to that noted in children, with the prevalence being the lowest at the same value of MUIC (around 200 μg/l) (Figure 2B). Other studies have also reported similar results in pregnant women (28–30). The relation between them could be explained from an etiological aspect. However, although the tendency of the “U” curve is no doubt when MUIC is particularly high, a few points may not fit well with the curve, sometimes, they might be higher or even lower. This wave-like rise at the high side of the curve might be caused by four reasons: first, the thyroid atrophy and fibrosis following enlargement due to extremely high iodine intake occur in some of the subjects (31, 32); second, the wave-like rise might be caused by the less and less sample size from middle to the high end, although the total sample size is huge, it is uneven between groups; third, as the extremely skewed distribution of urinary iodine, logarithm transformation, and grouping analysis methods are normally used to correct the “U” curve, but sometimes the curve is not very symmetrical, with a right tail; last, the sensitivity is different of individual suffered high iodine damage. Therefore, possibly, these points may not be outliers. This phenomenon could also be seen in other published articles (33, 34). Although these points weaken the stability and symmetry of the curve, they were valuable and made the results more reasonable and meaningful. In the fitting model of MUIC with the prevalence of goiter in children, the points were basically around the fitting curve, and the *R*^2^ was satisfied with the value around 0.8. In pregnant women, it does not fit as good as in children, but the result was also acceptable. The findings in children and pregnant women of this study provided additional evidence to support the range of MUIC in children (100–299 μg/l) and pregnant women (150–249 μg/l) as the cut-off values of these two indicators proposed in the 2007 criteria (2).

We evaluated the IDD elimination status in China using the 2018 China National IDD Surveillance data by four different methods. The 2007 criteria were recommended to evaluate the IDD elimination status at the national level, and in China, PHCAIS, MUIC in children and pregnant women, and the programmatic indicators at the national level were all above their cutoffs proposed in the 2007 criteria (2). To further assess the IDD elimination in local areas, the 2007 criteria were creatively applied for evaluation in individual provinces, municipalities, and counties in this study. The IDD elimination did not reach the criteria in all provinces, municipalities, and counties. The influencing factor was mainly the MUIC in pregnant women. In most areas under elimination in China, MUIC in pregnant women was slightly lower than 150 μg/l. However, the international organization mentioned that where USI has been effective for at least 2 years, with the PHCAIS more than 90%, it can be reasonably expected that the iodine needs of pregnant women are covered by their diet, and that the iodine stored in the gland is sufficient to ensure adequate hormone synthesis and secretion (35). The sustainable elimination of IDD is a long-term goal. Improvement in technical and programmatic indicators has implications for assessing programs and maintaining the sustainability of the elimination. In the 2007 criteria, the programmatic indicators cover 10 framework aspects which are basal and general requirements but do not have details for every aspect. To overcome these shortcomings, China established its system of programmatic indicators based on the 2007 criteria. The comparison with the 2007 criteria showcased the characteristics of the Chinese programmatic indicators (Supplementary Table 3). A scoring system is more applicable in China. It facilitates strengthening the capacity of teams for IDD prevention and control. Chinese programmatic indicators may provide useful information for the update of the 2007 criteria.

The 2018 China National IDD Surveillance data have strengths for the evaluation of IDD elimination, including the huge sample size and national representativeness. There are a few limitations to this study. Although we discussed the possible reasons for the high MUIC in children in areas with PHCAIS <90% in China, no corresponding investigation such as a detailed questionnaire survey on dining in school or processed food adding iodized salt was conducted in this study. Further research should focus on this phenomenon in the areas with low PHCAIS and high frequency of dining out and consuming processed food adding iodized salt in China. In addition, children with thyroid disease were not excluded in this research, and individual thyroid diseases in pregnant women were not analyzed separately. Last, although the polynomial regression model may be affected by potential “outliers,” information was still provided for reference.

In conclusion, IDD elimination has been essentially achieved in China evaluated by the 2007 criteria and the Chinese criteria; based on the 2018 China National IDD Surveillance data, recommendations and suggestions for revision of the 2007 criteria were made concerning adding the prevalence of goiter (<5%), keeping the ranges of MUIC (100–299 μg/l) in children and MUIC (>150 μg/l) in pregnant women, and the requirement of PHCAIS > 90%, especially for pregnant women. This study has importance and implications for providing evidence for the revision of international and domestic guidelines for IDD elimination and thus promoting global public health.

## Data Availability Statement

The datasets presented in this article are not readily available because they are the national surveillance data. Requests to access the datasets should be directed to Center for Endemic Disease Control, Chinese Center for Disease Control and Prevention.

## Ethics Statement

The study was conducted according to the guidelines of the Declaration of Helsinki and approved by Ethics Committee of Harbin Medical University, the code is ZZXM2018011. Written informed consent to participate in this study was provided by the participants' legal guardian/next of kin.

## Author Contributions

PL conceived and designed the study. LF wrote the original draft. FM, QS, and YZ performed the statistical analysis. All authors contributed to the acquisition, analysis, interpretation of the data, and approved the final version of the manuscript.

## Funding

This study was supported by the 2019 National Health Standards Project (20190502) and in part supported by the Natural Science Foundation of China (NSFC 81773370 and NSFC 82173638).

## Conflict of Interest

The authors declare that the research was conducted in the absence of any commercial or financial relationships that could be construed as a potential conflict of interest.

## Publisher's Note

All claims expressed in this article are solely those of the authors and do not necessarily represent those of their affiliated organizations, or those of the publisher, the editors and the reviewers. Any product that may be evaluated in this article, or claim that may be made by its manufacturer, is not guaranteed or endorsed by the publisher.

## Supplementary Material

Supplementary Figure 1 The correlation of goiter prevalence in provinces by B-ultrasound and by palpation (the Chinese IDD surveillance data from 1997 to 2002).

Supplementary Table 1 Classification of salt consumed in China based on iodine content.

Supplementary Table 2 Programmatic indicators for monitoring progress toward sustainable elimination of iodine deficiency disorders (IDDs) in China.

Supplementary Table 3 The comparison between the 2007 criteria and the Chinese criteria.
