# Supplementary figures and images for: Assessment of Sustainable Elimination Criteria for Iodine Deficiency Disorders Recommended by International Organizations
Source: Front Nutr. 2022 Apr 13;9:852398. doi: 10.3389/fnut.2022.852398 (PMC9043767; doi:10.3389/fnut.2022.852398)

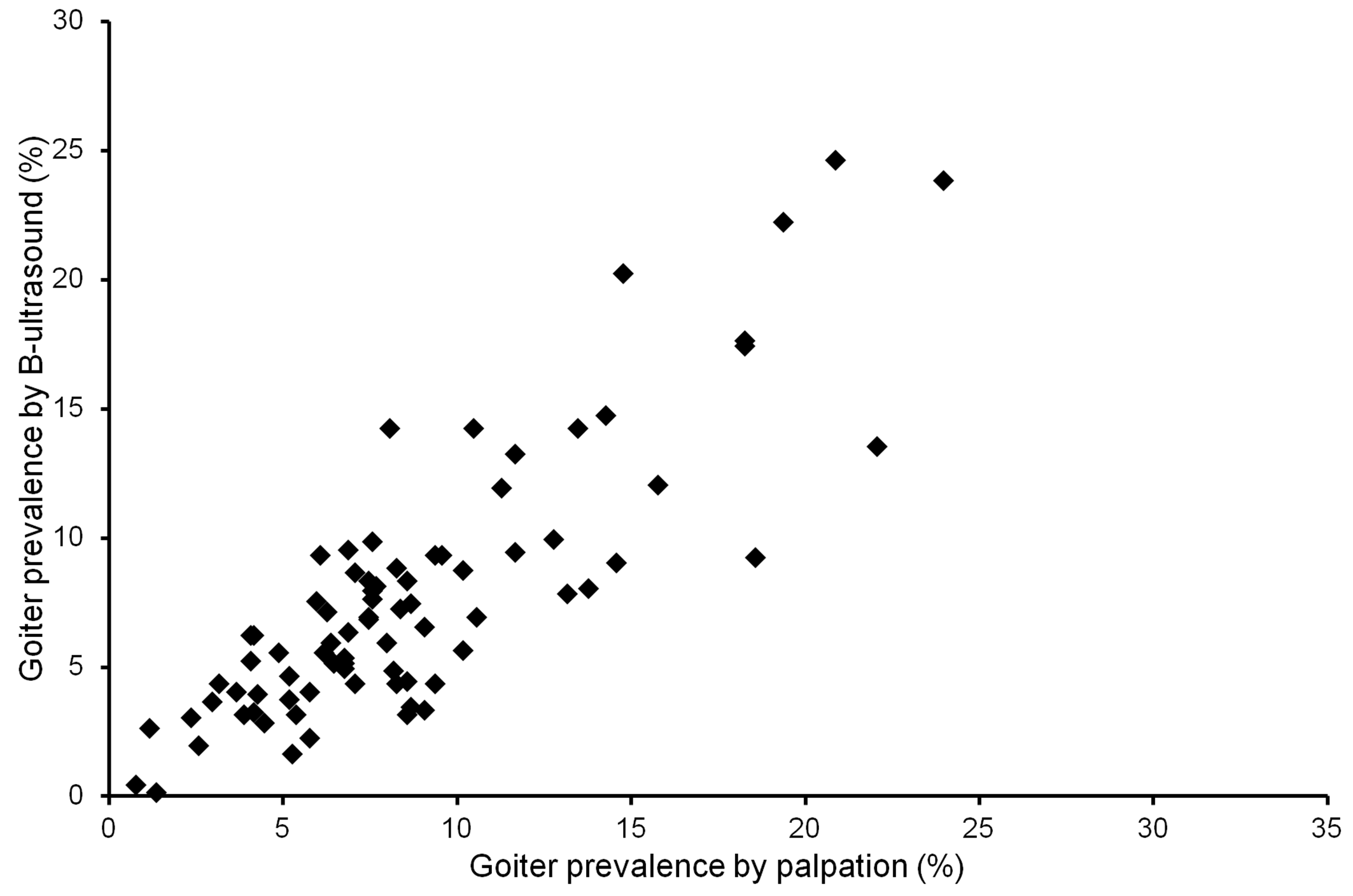

Supplement: Supplementary Figure 1 — The correlation of goiter prevalence in provinces by B-ultrasound and by palpation (the Chinese IDD surveillance data from 1997 to 2002). [file Image_1.TIF]
