# Supplementary material for: Assessment of Sustainable Elimination Criteria for Iodine Deficiency Disorders Recommended by International Organizations
Source: Front Nutr. 2022 Apr 13;9:852398. doi: 10.3389/fnut.2022.852398 (PMC9043767; doi:10.3389/fnut.2022.852398)
Supplement: Supplementary Table 1 — Classification of salt consumed in China based on iodine content. [file Table_1.DOCX]

**Supplementary Table 1.** Classification of Salt Consumed in China Based on Iodine Content

| Standard Content  of Iodine in Salt | Types of Salt Consumed(mg/kg) | | | |
| --- | --- | --- | --- | --- |
|  | Non-iodized | Low-iodized | Qualified iodized | High-iodized |
| 25 mg/kg | <5.0 | 5.0–17.9 | 18–33 | >33 |
| 30 mg/kg | <5.0 | 5.0–20.9 | 21–39 | >39 |
| 25 mg/kg for general population | <5.0 | 5.0–17.9 | 18–33 | >33 |
| 30 mg/kg for pregnant women | <5.0 | 5.0–20.9 | 21–39 | >39 |
