# Supplementary material for: Assessment of Sustainable Elimination Criteria for Iodine Deficiency Disorders Recommended by International Organizations
Source: Front Nutr. 2022 Apr 13;9:852398. doi: 10.3389/fnut.2022.852398 (PMC9043767; doi:10.3389/fnut.2022.852398)
Supplement: Supplementary Table 2 — Programmatic indicators for monitoring progress toward sustainable elimination of iodine deficiency disorders (IDDs) in China. [file Table_2.DOCX]

**Supplementary Table 2.** Programmatic Indicators for Monitoring Progress towards Sustainable Elimination of Iodine Deficiency Disorders (IDD) in China

| **Index** | **Details** | **Evaluation Criterion** | **Method** | **Scores** |
| --- | --- | --- | --- | --- |
| **Organization and leadership (Total score:22)** | Management by the government or other national entity (council or committee) | IDD prevention and control is incorporated into the **Governmental** Management Objectives: 2 points, or into the **Sectional** Objectives: 1 point;  the responsibilities of relevant governmental departments are clarified: 2 points. | Referring to the relevant governmental or sectional documents or commitment of responsibility | 5 |
|  | Increasing the capacity for IDD prevention and control teams | The number of staff is adequate for IDD prevention and control: 3 points. Staff members attended superior training for the past three years: 5 points, two years: 3 points, and one year: 2 points. | Reviewing relevant records. | 8 |
|  | Funding for IDD prevention and control | Enough funds were allotted for the prevention and control of IDD during the last three years: 3 points per year. | Reviewing allocation documents and receipts from the financial department | 9 |
| **Surveillance and prevention and control measures of IDD (Total score:32)** | Carrying out IDD surveillance | Observing the incidence of IDD during the last three years: 4 points per year. | Reviewing relevant records | 12 |
|  | Submitting IDD surveillance data and reports in time | Submitting IDD surveillance data and reports in time during the past 3 years: 2 points per year. | Reviewing relevant documents | 6 |
|  | Providing IDD surveillance results to the relevant departments on time | Providing the local governments and relevant departments with IDD surveillance reports during the past 3 years: 1 point per year | Reviewing relevant documents | 3 |
|  | Evaluating IDD progress independently or dependently | Evaluating IDD incidence in the last three years: 2 points. | Reviewing relevant documents | 2 |
|  | Participating in the quality control assessment of laboratory on a national or provincial level | Assessment of laboratory quality in the past three years: all passed, 6 points; not participating or partial pass: 3 points, all failed: 0 points. | Reviewing the relevant documents or laboratory assessment notifications | 6 |
|  | Implementing the provision of the emergency iodine supplement | Providing the emergency iodine supplement (iodized oil pill) to the target population in time in the past three years: 1 point per year;  no need for additional iodine supplementation: 1 point per year. | Reviewing iodine supplement records | 3 |
| **Supervision of Iodized Salt (Total score:20)** | Managing the production and circulation of iodized salt | Supervising the salt production and circulation in accordance with the relevant law: 2 points per year. | Reviewing relevant planning documents and summaries | 6 |
|  | Managing the salt industry and market (iodized salt supplement and non-iodized salt investigation) | Satisfactory consumption rates of qualified iodized salt consumption in terms of population status: 3 points per year;  No illegal cases or the illegal cases were effectively handled: 5 points. | Reviewing surveillance reports and documents of yearly illegal cases | 14 |
| **Health education (Total score:26)** | Providing education and promoting IDD control | Providing health education through the use of television programs or broadcasts: 5 points; through new media: 5 points. | Reviewing records of programs that had been broadcasted in the last three years | 10 |
|  | Providing health education in primary and middle school | Incorporating IDD health education into the Teaching Plan: 1 point per school; the maximum is 5 points for 5 schools. | Reviewing relevant documents | 5 |
|  | Providing fixed educational content | Setting the fixed education board in two and above sites per township (except for poster): 1 point; the maximum, 5 points for 5 townships. | Field investigation | 5 |
|  | Organizing activities on 5.15 IDD Day | The organization of educational activities by multiple departments in the past three years: 2 points per year; by the health department alone: 1 point per year. | Reviewing activity notifications, summaries, photographs, etc. | 6 |
| Total | | | | 100 |
