# Supplementary material for: Assessment of Sustainable Elimination Criteria for Iodine Deficiency Disorders Recommended by International Organizations
Source: Front Nutr. 2022 Apr 13;9:852398. doi: 10.3389/fnut.2022.852398 (PMC9043767; doi:10.3389/fnut.2022.852398)
Supplement: Supplementary Table 3 — The comparison between the 2007 criteria and the Chinese criteria. [file Table_3.DOCX]

**Supplementary Table 3.** The Comparison between the 2007 Criteria and the Chinese Criteria

| **Programmatic indicators in the 2007 Criteria** | **Programmatic indicators in the Chinese Criteria** | **Comparison** |
| --- | --- | --- |
| 1. Presence of a national multi-sector coalition responsible to the government for the national programme for the elimination of IDD with the following characteristics:  –National stature;  –All concerned sectors, including the salt industry, represented, with defined roles and responsibilities;  –Convenes at least twice yearly. | Management by the government or other national entity (council or committee) | Lack of rules for convenes in the latter. But in fact, it is hold once or above in China. |
| 2. Demonstration of political commitment as reﬂected by:  – Inclusion of IDD in the national budget (either as specific programme funds or through inclusion in existing programme funds) particularly with regard to procurement and distribution of KIO_3_. | Funding for IDD prevention and control. | Basically the same. |
| 3. Enactment of legislation and supportive regulations on universal salt iodization, which establishes a routine mechanism for external quality assurance. |  | The legislation of USI is not mentioned in this criteria, however, it has been legislated by another legislation since 1994, and will be updated recently. |
| 4. Establishment of methods for assessment of progress in the elimination of IDD as reﬂected by:  – Reporting on national programme progress every three years. | Evaluating IDD progress independently or dependently | Basically the same. |
| 5. Access to laboratories as defined by:  – Laboratories able to provide accurate data on salt and urinary iodine levels and thyroid function. | Participating in the quality control assessment of laboratory on a national or provincial level. | Basically the same. |
| 6. Establishment of a programme of education and social mobilization as defined by:  – Inclusion of information on the importance of iodine and the use of iodized salt, within educational curricula. | Providing education and promoting IDD control  Providing health education in primary and middle school  Providing fixed educational content  Organizing activities on 5.15 IDD Day | The latter also has 5.15 IDD Day every year. |
| 7. Routine availability of data on salt iodine content as defined by:  – Availability at the factory level at least monthly, and at the household level at least every five years. | Providing IDD surveillance results to the relevant departments on time | The factory iodine level is supervised by the producer, the market level is supervised by the Market supervisor bureau and the household iodine level is surveyed and available every year in the latter. |
| 8. Routine availability of population-based data on urinary iodine every five years. | Carrying out IDD surveillance;  Submitting IDD surveillance data and reports in time;  Providing IDD surveillance results to the relevant departments on time; | The latter not only provide the IDD surveillance data including urinary iodine, it also stipulate how often to carry out the IDD surveillance and data sharing. |
| 9. Demonstration of ongoing cooperation from the salt industry as reﬂected by:  – Maintenance of quality control measures and absorption of the cost of iodate/iodide. | Managing the production and circulation of iodized salt  Managing the salt industry and market (iodized salt supplement and non-iodized salt investigation) | Contents are basically the same. |
| 10. Presence of a national database for recording of results of regular monitoring procedures which include population-based household coverage and urinary iodine (with other indicators of iodine status and thyroid function included as available). | Carrying out IDD surveillance;  Submitting IDD surveillance data and reports in time;  Providing IDD surveillance results to the relevant departments on time | Contents are basically the same. |
|  | Implementing the provision of the emergency iodine supplement;  Increasing the capacity for IDD prevention and control teams | The latter also includes the emergency iodine supplement and increasing the capacity for IDD prevention and control teams. |
